# The Impact of Type 1 Interferons on Alveolar Macrophage Tolerance and Implications for Host Susceptibility to Secondary Bacterial Pneumonia

**DOI:** 10.3389/fimmu.2020.00495

**Published:** 2020-03-20

**Authors:** Emma Connolly, Tracy Hussell

**Affiliations:** Lydia Becker Institute of Immunology and Inflammation, The University of Manchester, Manchester, United Kingdom

**Keywords:** type I IFN, trained immunity, alveolar macrophage, lung viral infection, secondary bacterial pneumonia, epigenome, tolerance

## Abstract

That macrophages adapt to environmental cues is well-established. This adaptation has had several reiterations, first with innate imprinting and then with various combinations of trained, tolerant, paralyzed, or primed. Whatever the nomenclature, it represents a macrophage that is required to perform very different functions. First, alveolar macrophages are one of the sentinel cells that flag up damage and release mediators that attract other immune cells. Next, they mature to support T cell priming and survival. Finally they are critical in clearing inflammatory immune cells by phagocytosis and extracellular matrix turnover components by efferocytosis. At each functional stage they alter intrinsic components to guide their activity. Training therefore is akin to changing function. In this mini-review we focus on the lung and the specific role of type I interferons in altering macrophage activity. The proposed mechanisms of type I IFNs on lung-resident alveolar macrophages and their effect on host susceptibility to bacterial infection following influenza virus infection.

## Introduction

Bacteria entering the respiratory tract are generally tolerated well in healthy adults and their growth contained by the host commensal microbiome, antimicrobial peptides, phagocytic cells (predominantly macrophages), mucus entrapment, and ciliary clearance. Some bacteria associated with respiratory tract infections are part of the normal microbiome in health, such as *Streptococcus pneumoniae, Haemophilus influenza*, and *S. aureus* (1–3). However, severe consequences arise when the lung microenvironment is perturbed in some way. Perturbations can include underlying congenital abnormalities (e.g., primary ciliary dyskinesia), underlying chronic disease (e.g., asthma, chronic obstructive pulmonary disease, cystic fibrosis, idiopathic pulmonary fibrosis), the effect of the aging process, the premature lung and previous severe infections (4). In all cases, the outcome depends on the severity of the perturbation, the rate of bacterial growth, and whether the bacterium is contained in the airspaces or invades the lung tissue and systemic circulation.

Containment of bacteria relies on effective physical and chemical barriers, but also a timely immune response. Any delay in immunity allows the growth of bacteria to an over-whelming level. It is interesting to note that conditions associated with bacterial out-growth occur in situations where the lung has a heavy infiltration of the very cells (macrophages and neutrophils) required to clear the micro-organism, which suggests they are not functioning properly (5). The function and phenotype of any immune cell is influenced by the local microenvironment and the needs of the tissue at that time. We referred to this adaptation as “innate imprinting” in 2004 (6) that was superseded by the term “trained immunity” (7–10). However, the terminology continues to evolve and now trained immunity represents a “primed” state that is beneficial, whereas the more immune paralyzed state (as observed following viral infection of the lung) is referred to as a “tolerant” state. Trained/tolerant innate immunity is important in health, disease and disease resolution. The molecular mechanisms of trained immunity in health have been described extensively elsewhere (11). Here we will describe how alveolar macrophages are tolerised during and following inflammation with a specific emphasis on the role of type I interferons (type I IFNs).

## Do Myeloid Cells Adapt?

Specificity and adaptation were once the hallmark of adaptive immunity alone. However, epidemiological studies as early as 1946 recognized that the *Mycobacterium tuberculosis* vaccine, BCG, also protected against childhood mortality caused by antigenically indistinct organisms, suggesting “adaptation” of cells of the innate, rather than adaptive, immune system (12, 13). Since then more recent studies have shown that innate immune cells can display adaptive characteristics (11). In terms of generating a specific response, it could be argued that pattern recognition receptors (PRRs), expressed by innate immune cells, confer specificity. PRRs are germline-encoded receptors and include the toll-like receptors (TLRs), RIG-I-like receptors (RLRs), NOD-like receptors (NLRs), and C-type lectins (14), among others. These receptors vary widely in the ligands that they bind to, allowing them to detect a substantial range of molecular patterns, known as pathogen- and damage-associated molecular pathogens (PAMPs and DAMPs, respectively) (15). This activates both divergent and convergent downstream signaling pathways enabling a tailored response to a specific pathogen (14). Furthermore, it is now recognized that innate immune cells, for example myeloid cells (7, 8, 16), NK cells (17, 18) and epithelial cells (19), can acquire “memory”, characterized as a heightened and quicker response upon re-exposure to a pathogen. Innate immune memory is well-defined in organisms that lack an adaptive immune system, including plants and invertebrates (20, 21). This is more controversial in vertebrates, partly due to the relatively short half-life of innate cells, which in the case of monocytes can be up to 1 day in the circulation (22). However, the presence of innate immune memory in monocytes has been observed for up to 3 months (13) and for macrophages 6 months or more (23). This innate immune memory or trained immunity likely serves as an evolutionary survival advantage with the innate immune system primed to combat a secondary pathogen encounter (11). However, training can lead to deleterious consequences if the outcome is a macrophage that is tolerant to stimulation. A slower macrophage response likely protects the host from further tissue damage, prioritizes a reparative state and prevents the development of autoimmunity. In the case of severe influenza virus infection, upon resolution macrophages are unable to respond quickly enough to curtail bacterial load leading to complications of secondary pneumonia (24).

## Type I IFNs

**Figure 1:**
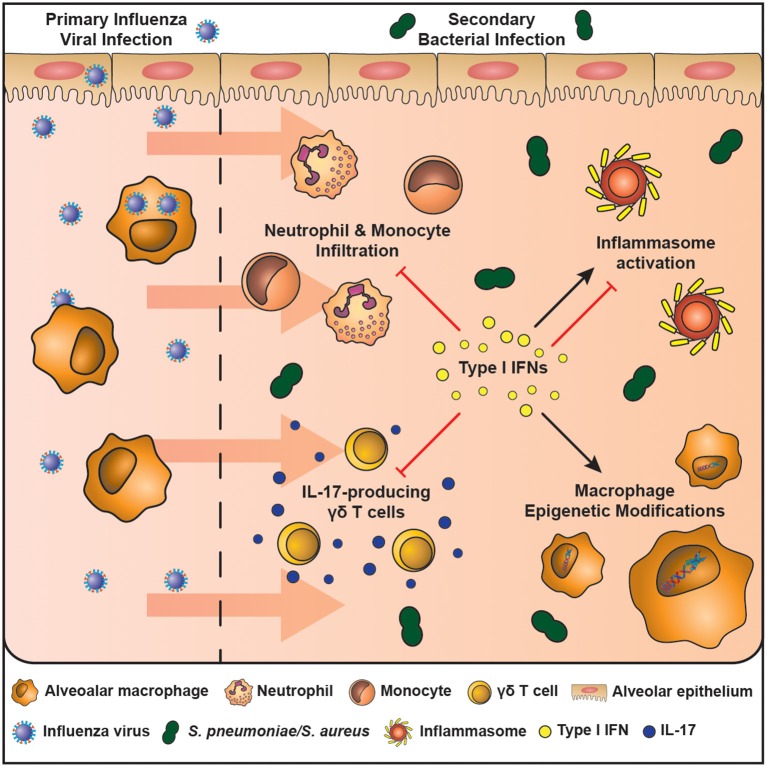
The mechanisms of enhanced host susceptibility to secondary bacterial infection by type I IFNs. Airway epithelial cells and alveolar macrophages are cells of the innate immune system that are at the first line of defense against infection in the airspaces. The influenza virus activates pattern recognition receptors expressed by airway epithelial cells and macrophages and leads to the production of type I IFNs, which are crucial in combating the infection. However, type I IFNs also induce an immunosuppressive state in the resolution phase of infection that enhances host susceptibility to secondary bacterial infection. These mechanisms include: (1) inhibition of IL-17-producing γδ T cells, (2) Induces macrophage epigenetic modifications, (3) Induces or inhibits inflammasome activation in a context-dependent manner, (4) Inhibits neutrophil and monocyte infiltration. These mechanisms result in a lung environment ill equipped to fight an increasing bacterial burden.

There are many mechanisms associated with susceptibility to bacterial complications following lung viral infection. However, type I interferons (IFNs) stand out as particularly important as they directly impair, or lead to downstream consequences affecting, bacterial clearance (Figure 1) (25–30). All three types of interferons (Types I – III) play a major role in innate and adaptive immunity (14). Of the eight (-α, -β, -δ, -ε, -ζ, -κ, -τ, and –ω) type I IFNs, the -α, -β forms, which bind to the IFNAR receptor complex (IFNAR1 and IFNAR2), have received the most attention with regards to lung viral infection (32). Receptor binding recruits janus kinase 1 (JAK1) and tyrosine kinase 2 (TYK2) that leads to the phosphorylation of Signal Transducer and Activator of Transcription (STATs). Phosphorylation leads to homodimers and heterodimers; the precise combination dictating the final transcriptional outcome. STAT 1 and 2 heterodimers bind to IRF9 to form the ISG (Interferon Stimulated Gene) factor 3 complex−9 (33, 34). Type I IFNs also activate the p38-associated MAPK (mitogen-activated protein kinase pathway) (35). Type I IFNs have a myriad of functions in the lung where they are both crucial for the clearance of viral infection and resolution of inflammation. However, it is these diverse functions that are thought to contribute to host susceptibility to bacterial infections following viral infection.

## Type I IFNs and Host Susceptibility to Secondary Bacterial Infection

The contribution of type I IFNs to host susceptibly to bacterial infection is well-established (Table 1). In 2001, Biron's group defined a role for IFN α/β in viral-induced sensitization to bacterial products (36). Viral mimics, such as Poly I:C, also impair anti-bacterial immunity by induction of type I IFNs (37). Since then the field has expanded rapidly to show that type I IFNs decrease neutrophil chemoattractants (CXCL1/2) (25, 26), reduce IL-17 producing γδ T cells (27), and impair CCL2- mediated recruitment of macrophages following viral infection (28). Furthermore, depending on the context type I IFNs can promote or inhibit NLRP3 inflammasome activation, causing either an increase in IL-1β that limits γδ T cell activity with subsequent susceptibility to *S. pneumoniae* (27) or decreases IL-1β production enhancing susceptibility to *S. aureus* (29, 30), respectively. Additionally, type I IFN induced by viral infection alters cellular metabolism that may favor bacterial replication, uptake and adhesion (38). These mechanisms have been reviewed extensively elsewhere (39).

**Table 1:** The effects of viral—induced type I IFN on the inflammatory response to secondary bacterial infections.

	**Primary viral infection**	**Secondary bacterial infection**	**Type I IFN-mediated effects (↓= decreased; ↑= increased)**	**References**
Inflammatory response	H1N1 influenza A/PR/8/34 virus (PR8)	Type 3 S. *pneumoniae*	↓ Neutrophil chemoattractants (CXCL1/2)	(25, 26)
	Influenza virus A/X31 (H3N2)	Type 3 S. *pneumoniae*	↓ IL-17-producing gamma delta T cells	(27)
	H1N1 influenza A/PR/8/34 virus (PR8)	Strain P1121, S. *pneumoniae*	↓ CCL2- mediated recruitment of macrophages following viral infection	(28)
	Influenza virus A/X31 (H3N2)	Type 3 S. *pneumoniae*	↑ NLRP3 inflammasome activation	(27)
	Influenza A/PR/8/34 H1N1	Methicillin-sensitive *S. aureus*	↓ NLRP3 inflammasome activation	(29, 30)
Epigenetic modifications	H1N1 influenza A/PR/8/34 virus (PR8)	Type 3 S. *pneumoniae*	↑ Production of the methyltransferase Setdb2 ↑ H3K9me3 chromatin marks at the CXCL1 promoter ↓ Neutrophil Infiltration	(31)

The immune suppressive outcome of enhanced type I IFNs is exemplified by strategies to inhibit its action. Inhibition of IFN receptor I- and III-associated TYK2 restores anti-bacterial immunity in a human *ex vivo* lung co-infection model (40). An absence of STAT2 that is downstream of IFN-αR 1/2 makes influenza infection more severe, but prevents the development of secondary bacterial pneumonia (41). Furthermore, blocking Toll-like receptor 4 (TLR4) after influenza virus infection decreases bacterial growth by reducing IFNβ (26). Type I IFN induction may also contribute to the risk of bacterial infection following the administration of anesthetics prior to surgery. Infectious risk due to the immune modulatory effects of anesthetics delays surgical procedures in patients suspected of a respiratory infection. However, not all anesthetics cause this problem (42) and halothane actually reduces bacterial burden in influenza infected mice by decreasing type I IFN in the mouse lung (43). These observations suggest that type I IFN-induced tolerance following severe lung viral infection, although beneficial in limiting excess tissue damage and restoring tissue to homeostasis, results in a macrophage unable to deal with a growing bacterial burden.

## Type I IFNs and the Restoration of the Steady State

Type I IFNs are directly involved in important processes necessary to restore the lung to health. A reduction of inflammatory responses during apoptotic cell clearance is critical to prevent autoimmunity to self-antigens. Type I IFN receptor signaling induces suppressor of cytokine signaling (SOCS) 1 and 3 activation during efferocytosis of apoptotic cells by the receptor tyrosine kinase AXL (44). The combination of AXL and IFNAR1 signaling causes reduced macrophage responses and subsequent bacterial complications (45, 46). Furthermore, macrophages are also “tolerised” during the uptake of extracellular matrix turnover by-products; again an important function to restore homeostasis (47). The glycosaminoglycan, hyaluronan for example, is a prevalent extracellular matrix component in the lung (48), but it suppresses alveolar macrophage activity and is maintained at a higher level following resolution of a severe viral infection (49). Similarly, versican, a chondroitin sulfate proteoglycan, is expressed at low levels in the healthy lungs, but upregulated by TLR agonists LPS and Poly I:C and requires TLR, TRIF and type I IFN signaling. In turn versican up-regulates IL-10 and IFNβ, leading to an immune suppressive state (50). Therefore, repairing the damaged lung and restoring the steady state, impairs inflammation and involves type I IFNs. This raises the possibility that trained immunity in macrophages simply represents a change in function from inflammation to homeostatic maintenance.

## Epigenetic Modifications in Trained Immunity

The longevity of alterations in lung immunity following severe viral infection is surprising considering the relatively short life of innate immune cells. However, alveolar macrophages in particular, turnover relatively slowly in health (51). Of particular relevance to the altered reactivity of alveolar macrophages, is their re-wiring by epigenetic changes (52). Epigenetic changes are mediated by (micro) miRNAs, DNA methylation, and histone modifications, amongst others and regulate chromatin accessibility (53). Chromatin accessibility determines which genes are visible and therefore impacts on cellular signaling and gene expression.

Monocyte/Macrophage adaptation is accompanied by fundamental epigenetic changes (54, 55) and is often associated with alterations in cellular metabolism (56, 57). Trained monocytes, producing excess TNFα and IL-6 protect RAG-/- mice (lacking functional T and B lymphocytes) against reinfection with *Candida albicans* due to stable histone trimethylation at H3K4 (8). Candida binding to Dectin-1 causes stable changes in histone trimethylation at H3K4 and increases the immune responsiveness of monocytes (8). Similarly, chromatin modifications by BCG vaccination provide protection to unrelated infections (13). Tolerance induction in macrophages cultured with LPS results in methylation at H3K9me2 and H3K9me3 and protects against S. *aureus* infection (58). Looking beyond pro-inflammatory processes, it is clear that in tolerised macrophages not all genes are repressed in all circumstances. For example, LPS-stimulation of murine macrophages *in vitro* represses pro-inflammatory genes, but enhances genes encoding anti-microbial effector proteins (16). However, this is often not the case *in vivo*, where reduced anti-bacterial immunity and macrophage effector function are observed following viral infection. This discrepancy, represents an opportunity since it suggests that some stimuli lead to a different macrophage outcome. A recent study of influenza infection followed by a S. *pnemoniae* strain lacking the major virulence factor pneumolysin, shows that not all macrophages are affected equally and that long term epigenetic changes differ between recruited and resident macrophages (59). Understanding how to achieve a bactericidal vs. an anti-inflammatory macrophage outcome could provide strategies to combat post-viral bacterial pneumonia.

## Type I IFN-Induced Epigenetic Modifications

Type I IFN modification of the epigenetic landscape is mostly via their regulation of interferon-stimulated genes (ISGs) (60, 61). ISGs encode a wide range of proteins that restrict viral infection and spread, including inhibition of viral transcription, translation and replication, the degradation of viral nucleic acids and the alteration of cellular lipid metabolism (62, 63). Approximately 2,000 human and mouse ISGs have been identified and cataloged in the Interferome database (64). All classes of IFNs have overlapping ISGs (65, 66) and so it remains unclear how ISGs are regulated in order to produce a unique and tailored response to a given pathogen. Epigenetic modifications are proposed as one mechanism by which ISG transcription can be context specific (65). The ISGs induced may depend on the cell type, the exposure of the cell to other stimuli, such as PAMPs or DAMPs, or the strength and duration of the interferon stimulus. All these variables may affect the chromatin landscape and provide another level of ISG regulation to different environmental cues. Evidence shows that enhanced transcription of ISGs upon re-stimulation is not due to increased expression of the required transcription factors or IFN signaling molecules, but rather as a result of altered chromatin marks at ISG promoters, thereby priming or repressing certain ISGs. Of the 1,000 s of ISGs known, only half are reported to become primed or display “memory” upon restimulation (61). Other inflammatory factors present in the microenvironment also affect the profile of ISGs available. For example, in response to LPS, type I IFNs prevent the silencing of inflammatory genes driven by prior TNF exposure of macrophages. This is mediated by an altered chromatin state, with increased recruitment of H4ac and H3K4me3 histone marks that are generally associated with transcriptional activity, and increased chromatin accessibility at tolerised genes (60). In addition to driving alterations in the epigenome, type I IFNs can also be regulated by epigenetic modifications. For example, miR146a (67), and miR26a (68) promote type I IFNs and reduce influenza infection in experimental models, whereas miR29a reduces IFNAR1 and has the opposite effect (69).

## Manipulation of the Epigenome to Reverse Tolerance in Macrophages

An important aspect of viral-induced macrophage tolerance to consider is whether it can be overcome or reversed in order to unleash the full inflammatory potential of macrophages and promote anti-bacterial responses. One possibility could be via manipulation of epigenetic changes. For example, histone modifications are reversible and therefore can be altered. The methyltransferase Setdb2 is an ISG that regulates the production of the neutrophil chemoattractant CXCL1. Deletion of Setdb2 decreases H3K9me3 chromatin marks, releases the CXCL1 promoter from inhibition, enhances airway neutrophil infiltration and reduces susceptibility to secondary *S. pneumoniae* (31). Furthermore, β-glucan can overcome the tolerised phenotype of macrophages following LPS exposure (70) and monocytes from experimental endotoxemia in healthy volunteers. This suggests that it is possible to improve the antibacterial function of macrophages. A tolerance phenotype is also observed in other cells. Airway epithelial cells, for example, are also refractory to TLR agonists following stimulation that can be restored by histone deacetylase inhibitors (71). Although not specifically identified to our knowledge, it would be interesting to determine whether these inhibitors could potentially reverse macrophage tolerance and reduce susceptibility to secondary bacterial infections.

## Type I IFN Treatment and the Prevention of Bacterial Super-Infections

The post-viral lung effects of type I IFNs span multiple bacterial species, including *Streptococcus pneumoniae* (25), *Pseudomonas aeruginosa* (72), *Staphylococcus aureus* (73) [including multi-drug resistant forms (41)] and *Escherichia coli* (74). Furthermore, the preceding viral infection need not be in the lung. For example, systemic Lymphocytic choriomeningitis Virus (LCMV) infection causes apoptosis of granulocytes in the bone marrow leading to reduced recruitment of neutrophils to the airways during Listeria *monocytogenes* or *S. aureus* infection (75). Therefore, manipulation of type I IFNs may represent a therapeutic option once bacterial complications arise following severe viral lung infection. Targeting of type I IFN responses is currently used in the treatment of several inflammatory and autoimmune diseases. For instance, IFNβ is an effective therapy for multiple sclerosis patients and IFNα has been approved for the treatment of hepatitis B and C (76). In contrast, the blockade of the type I IFN receptor with anti-IFNAR, has been an attractive therapeutic for autoimmune diseases including systemic lupus erythematosus (SLE) and rheumatoid arthritis, as these diseases are characterized by a profound IFN gene signature (77, 78). However, difficulties in developing effective therapies that target the type I IFN system relies upon selecting the specific type I IFN to administer or block, and the timing of drug delivery, which can lead to opposing outcomes. This is observed by the pro-inflammatory and immunosuppressive mechanisms that type I IFNs can generate in the tumor microenvironment. Although IFNα immunotherapy has proven effective in the treatment of hematological malignancies (79, 80), type I IFN treatment of solid tumors has shown less potential (81). Conversely, type I IFN inhibition can promote an anti-tumor responses by unleashing the inflammatory potential of exhausted T cells and removing the requirement for combinatorial immune checkpoint inhibitor immunotherapies (82). Further understanding of the roles of individual interferons in different inflammatory contexts and the divergent downstream signaling pathways they trigger is still required to generate effective treatment options. Currently, research is lacking for targeting type I IFNs to treat secondary bacterial pneumonia. However, studies suggest that targeting the epigenome of ISGs may be a more successful avenue of investigation. This would more likely limit potential negative side effects that may arise from removing type I IFNs themselves.

## Conclusion

Type I IFNs clearly play a central role in bacterial super infections following lung damage, particularly that caused by pulmonary viral infection. Here we have focused on the effect of, predominantly, influenza infection on macrophages. However, similar processes may exist following infection with other respiratory viruses, such as respiratory syncytial virus. Collectively, the evidence suggests that overcoming type I IFN driven immune suppression may be beneficial for viral-induced bacterial super infection. Anti-IFNAR (e.g., Sifalimumab) is already used in the treatment of SLE (83) and could be repurposed for post-viral lung conditions. However, any strategy would need to be carefully timed and type I IFN administration during influenza infection may enhance viral immunopathogenesis. Bacterial infections mostly arise when the bulk of viral titer has been eliminated. Sometimes there is a sufficient and visible window between viral infection and bacterial outgrowth that would allow timed treatment to be administered. However, ultimately the problem is dependent in the first place on the severity of the viral infection. Studies to date show that any strategy that reduces the impact of lung viral infection reduces the chances of developing subsequent bacterial complications. Vaccination would therefore still seem the best policy; as long as any attenuated forms do not induce excess type I IFNs themselves. Finally, we should remember that macrophages attune to the needs of the tissue. Their trained/tolerant/primed state is therefore not abnormal, but rather represents a macrophage that has to first inflame to recruit immune cells, then change to professionally instruct them and finally clear up the mess afterwards.

## Author Contributions

EC and TH drafted the manuscript, made substantial contributions to the conception and design of the work, approved the submitted version of the manuscript, and agreed to be accountable for all aspects of the work.

### Conflict of Interest

The authors declare that the research was conducted in the absence of any commercial or financial relationships that could be construed as a potential conflict of interest.
